# Performance of the Eight-item Informant Interview to Differentiate Aging and Dementia within a context similar to the Swedish primary healthcare sector: a systematic review of diagnostic test accuracy studies

**DOI:** 10.1080/02813432.2020.1844370

**Published:** 2020-11-20

**Authors:** Annsofie Svensson, Eva Granvik, Katarina Sjögren Forss

**Affiliations:** a Kunskapscentrum demenssjukdomar VE minnessjukdomar Skånes University Hospital Malmö, Sweden; b Faculty of Health and Society, Department of Care Science, Malmö University, Malmö, Sweden

**Keywords:** Dementia, diagnostic test accuracy, Eight-item Informant Interview to Differentiate Aging and Dementia (AD8), primary healthcare, screening, systematic review

## Abstract

**Objective:**

Dementia is a common but underdiagnosed health problem. Instruments developed for initial screening exist internationally but are not available within the Swedish primary healthcare sector. This systematic review aimed to evaluate the diagnostic test accuracy of the Eight-item Informant Interview to Differentiate Aging and Dementia in identifying symptomatic dementia within a context similar to the Swedish primary healthcare sector.

**Design:**

A systematic search was conducted in PubMed, CINAHL, PsycInfo, Cochrane Library and manually *via* reference lists up to November 2019. Eligibility criteria were the reporting of Diagnostic test accuracy outcomes for the Eight-item Informant Interview to Differentiate Aging and Dementia’s ability to identify dementia according to internationally approved criteria. The population of interest was selected within the community or primary care. QUADAS-2 was used for quality assessment, and data were analysed with a narrative approach.

**Results:**

Five studies with a total of 13,345 participants were included. With sensitivity (88–100%), specificity (67–91%), positive and negative predictive values (28–63%; 96–100%) respectively, the results show that the Eight-item Informant Interview to Differentiate Aging and Dementia has good ability to identify true positives, false negatives and predict low-risk dementia. That is, the Eight-item Informant Interview to Differentiate Aging and Dementia has a greater ability to predict people who are at risk of not having dementia than to correctly identify those at risk of having dementia within the target population.

**Conclusion:**

The results show that the Eight-item Informant Interview to Differentiate Aging and Dementia has the ability to identify persons with symptomatic dementia within the target population. Thus, an evaluation of its potential benefits should be considered and evaluated within the Swedish primary healthcare context.

**KEY POINTS**
- Dementia is a common but underdiagnosed health problem. Instruments developed for initial screening exist but are not available within the Swedish primary healthcare sector.
- We found that the Eight-item Informant Interview to Differentiate Aging and Dementia (AD8), has the ability to identify individuals with symptomatic dementia within the target population.
- The Eight-item Informant Interview to Differentiate Aging and Dementia (AD8), has the potential to increase the possibility for timely detection of individuals with symptomatic dementia.

## Introduction

In Sweden, an assessment for dementia is to be initiated by primary healthcare upon suspicion of dementia [1]. However, with an estimated prevalence of 150,000 persons with dementia, approximately 50% still remain unidentified [2,3]; this indicates difficulties in identifying symptomatic dementia. Though instruments developed for initial screening exist internationally, they are not available within the Swedish primary healthcare sector.

Missed or delayed evaluation and diagnosis may result in non-detection of treatable causes for cognitive impairment and reduce the patient’s opportunities for self-determination and involvement in decision-making for future planning and care [3,4]. Knowledge of a patient’s medical condition is essential to make an overall assessment and to provide person-centred care for both patients and their caregivers. Timely detection is consistent with good and safe healthcare [5] and is supported by the World Health Organisation’s seven-point dementia global action plan [6]. As the diagnosis itself is the key to customized help and support, being identified early in the disease process allows the patients, their caregivers and the healthcare system to work proactively [3,4].

In the absence of a cure, general population screening is not recommended. Instead, timely diagnosis can be facilitated by indicated screening [3], which in a Swedish primary healthcare context may be appropriate within, for example, annual controls at the general practitioner, health controls at the nurse practitioner in primary healthcare receptions for older adults, and in outreach programs for older adults within the community. As the purpose of a screening test is not to diagnose but to select those who would benefit from further diagnostic evaluation [7], this could provide valuable information to the often time-limited and complex decision-making process within primary healthcare [8,9].

In contrast to general population screening, which is performed in a preclinical asymptomatic phase [10], this type of screening is performed to identify persons already symptomatic. Thus, identification of dementia cannot be performed earlier than the person has symptoms, namely, when their cognitive functioning already has an impact on their everyday life [11]. In addition, cognitive functioning must be deterioration from the previous level. As self-reported cognitive decline is at risk of not being a reliable criterion [12], these kinds of instruments are informant-based with questions based on symptoms that may be a consequence of dementia. In contrast to the performance-based instruments recommended within the Swedish basic evaluation, whose diagnostic purpose is to objectively verify the symptoms [13–18].

In Sweden, the Cognitive Impairment Questionnaire (CIMP-QUEST) [19] is a well-known informant-based questionnaire, tested for validity and reliability to identify brain region-oriented symptomatology in patients with diagnosed mild cognitive impairment (MCI) and mild dementia. With 47 questions scored on a four-point scale, the CIMP-QUEST adds valuable information within the assessment and diagnostic process of dementia. However, too comprehensive to fulfil the characteristics as an initial screening tool. Another commonly known and used informant-based questionnaire in Sweden, even if no translation is tested for validity or reliability, is the Functional Activities Questionnaire (FAQ) [20]. The FAQ consists of ten questions scored on a six-point scale with a total of 30 points, developed to assess independence in activity of daily living. Thus, not suitable as an initial screening for dementia.

Two internationally known informant-based questionnaires, developed to screen for dementia by differentiating cognitive symptoms from normal aging, are the Informant Questionnaire on Cognitive Decline in the Elderly (IQCODE) [21] and the Eight-item Informant Interview to Differentiate Aging and Dementia (AD8) [22]. None of which are available with validity or reliability tested translation in Swedish. In its short version, the IQCODE consists of 16 questions scored on a five-point scale on the basis of current cognitive functioning compared to 10 years earlier. The total score of 16–80 is divided with 16 to a final score of 1–5. The diagnostic test accuracy (DTA) in identifying dementia among community-dwelling shows 80% sensitivity and 84% specificity [23].

The AD8 consists of eight Yes/No questions with a total score of 1–8 points, on the basis of cognitive functioning compared to 2 years earlier. In its original study [22], the AD8 showed a DTA of 85% sensitivity, 86% specificity and area under the curve (ROC) of 90% in discriminating non-dementia from dementia.

The AD8 has since its original study been validated or translated into multiple languages and is used in many countries worldwide [24–38]. The DTA of the AD8 has been evaluated with meta-analyses for the detection of both MCI and dementia in two systematic reviews towards different populations [39,40], the latest with a publication date of June 2018. In both, it was concluded that significant heterogeneity may have affected the validity of the summary findings.

The DTA of the AD8 is not affected by education level, gender or cultural aspects. It requires no formal training to administer, score or calculate. Moreover, it takes only 2–3 min to complete and can be filled in at home, by phone or during a visit [25]. Thus, it should be suitable as an initial screening instrument within the Swedish primary healthcare context.

To the best of our knowledge, no systematic review has been performed of the AD8’s DTA in identifying dementia in a non-selected population within both primary and community care. Therefore, the aim of this systematic review was to evaluate the DTA of the AD8 within a context similar to the Swedish primary healthcare sector.

## Material and methods

A systematic review was conducted. The methodological performance and reporting followed the guidelines according to the Centre for Reviews and Dissemination [41] and the Preferred Reporting Items for Systematic Reviews and Meta-Analyses of Diagnostic Test Accuracy Studies [42], respectively.

### Search strategy

A systematic search was performed in the databases PubMed, CINAHL, PsycINFO and Cochrane in June 2019 with an update in November 2019. The search strategy conducted in PubMed is presented in Appendix 1. Additionally, the reference lists of identified studies were manually searched. Search words were restricted to terms within Index test and Outcome. The limitation was only restricted to publication date August 2005, when the original study of the AD8 was published. Eligible studies of interest were studies reporting DTA (Outcome) in the detection of dementia (Reference standard) for the AD8 (Index test), with sensitivity, specificity, prevalence and used cut-off reported. Any reference standard internationally approved was accepted. The population of relevance was a consecutive or random sample within the community or primary care.

### Quality assessment

The assessment of quality was conducted according to the QUADAS-2 assessment tool [43]. In accordance with the QUADAS-2, each of the studies risk of bias within the four domains – (1) Patient selection, (2) Index test, (3) Reference standard and (4) Flow and timing – were assessed for low, unclear or high risk, as well as applicability concerns within the first three domains. The assessment was conducted by the first and second authors independently, and discrepancies were discussed and corrected in agreement with the third author.

### Data extraction

DTA outcome measures and study characteristics were extracted by the first author on the basis of eligibility criteria. DTA outcome measures as true and false positive and negative values, respectively, and positive and negative predictive values, and accuracy and likelihood ratio were calculated based on sensitivity, specificity and prevalence if not reported.

### Data synthesis

With regards to the applicability, the data were synthesised with a narrative approach though mapping, and a quick search showed no studies performed within a Swedish primary healthcare context.

## Results

**Figure 1.**
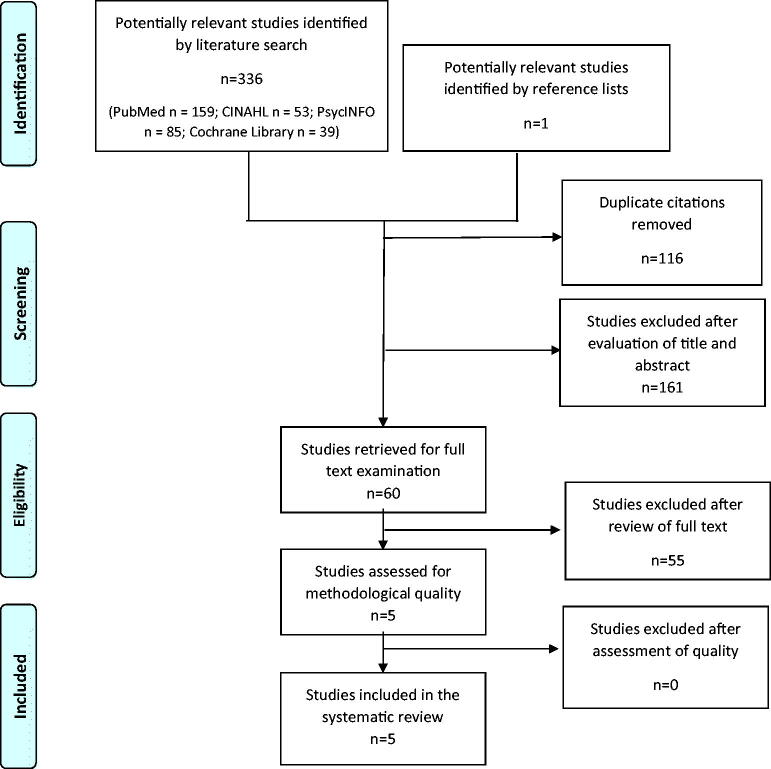
Flow chart detailing identification and selection of studies for inclusion in the review.

**Figure 2.**
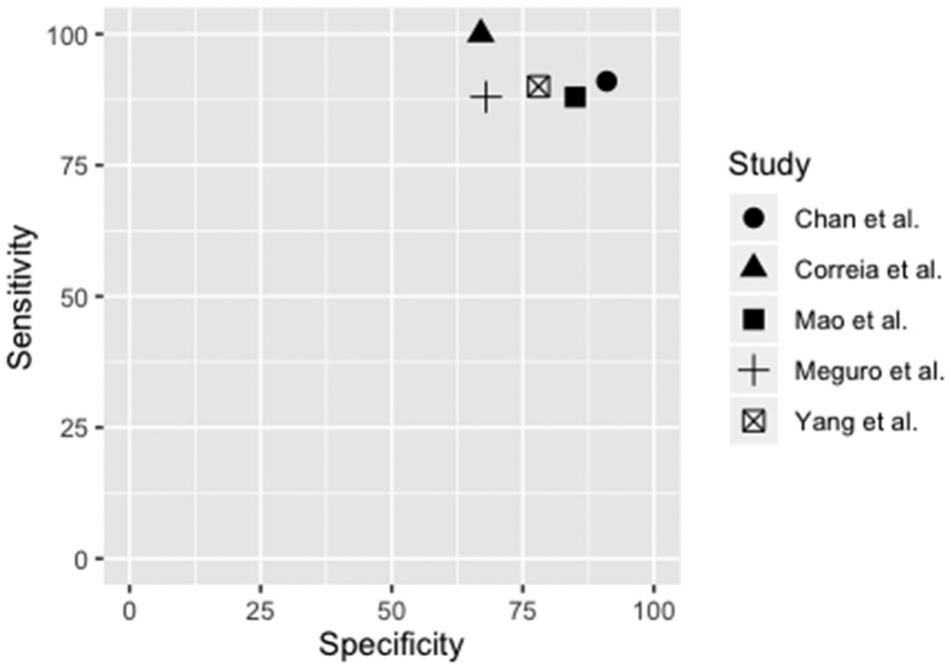
Illustration of sensitivity and specificity of included studies.

By using the key search descriptors, 336 studies were identified (Figure 1). One additional study was identified *via* a manual search of reference lists. After removing duplicates and assessing eligibility based on title and abstract, 60 studies were read in full text. Five studies met the inclusion criteria [32,36,44–46], that is, reporting of DTA outcome measures for the AD8 in the detection of dementia with cut-off reported and use of an internationally approved reference standard, as well as the population being sampled randomly or consecutively within the community or primary care (Appendix 2). Of those, four studies were written in English and one in Japanese, with the latter translated into Swedish by a professional Japanese/Swedish translator. DTA summaries of the AD8’s ability to identify dementia by included studies, along with study characteristics are presented in Table 1. The sensitivity and specificity of included studies, illustrated in Figure 2, were 88–100% and 67–91%, respectively, with a prevalence ranging from 8.9 to 22%. The positive and negative predictive values were between 28 and 63% and 96 and 100%, respectively. Positive and negative likelihood ratios were between 2.80 and 9.99 and 0.00 and 0.17, respectively. Accuracy was between 71 and 91%. For calculations see Appendix 3.

**Table 1.** Characteristics, accuracy summaries and summary of findings of included studies in AD8’s DTA for identifying dementia.

Study; year	Country; language of AD8	Setting	Size (*n*)	Age (mean)	Reference standard	AD8 cut-off	Prev.% (*n*)	Sens. (%)	Spec. (%)	TP (*n*)	FP (*n*)	FN (*n*)	TN (*n*)	PPV (%)	NPV (%)	LR+	LR−	Acc. (%)
Chan et al. [44]; 2016	Singapore; Chinese + Malay	Primary health care	309	≥60 (71.7)	DSM IV	≥3	14.2 (44/309)	91	91	40	24	4	241	63	98	9.99	0.10	91
Correia et al. [36]; 2011	Brazil; Brazilian-Portuguese	Community dwelling	109	≥65 (76.7)	DSM IV	≥3	13.8 (15/109)	100	67	15	31	0	63	33	100	3.03	0.00	72
Mao et al. [45]; 2018	Taiwan; Chinese	Community dwelling	10,340	≥65 (74.9)	NIA-AA	≥2	8.9 (917/10340)	88	84	804	1464	113	7979	35	99	5.66	0.15	85
Meguro et al. [32]; 2015	Japan; Japanese	Community dwelling	572	≥75 (80.1)	DSM IV	≥2	12.0 (69/572)	88	68	61	159	8	344	28	98	2.80	0.17	71
Yang et al. [46]; 2016	China; Chinese	Community dwelling	2015	≥65 (79.5)	NIA-AA	≥2	22.0 (444/2015)	90	78	398	339	46	1232	54	96	4.15	0.13	81
Summary of findings: (Mean)			13,345	76.6			14.2 (1489/13345)	91	78	1318	2017	171	9859	43	98	5.1	0.1	80

DTA: diagnostic test accuracy; DSM IV: Diagnostic and Statistical Manual of Mental Disorders; NIA-AA: National Institute on Aging and Alzheimer’s Association; Prev.: prevalence; Sens.: sensitivity; Spec.: specificity; TP: true positive; FP: false positive; FN: false negative; TN: true negative; PPV: positive predictive value; NPV: negative predictive value; LR+: positive likelihood ratio; LR−: negative likelihood ratio; Acc.: accuracy.

### Description of studies

The included studies were performed in Asia [32,44–46] and South America [36], with target populations recruited from the community [32,36,45,46] and primary healthcare [44] settings. Reference standards used were the National Institute on Aging and Alzheimer’s Association (NIA-AA) [45,46] and the Diagnostic and Statistical Manual of Mental Disorders (DSM IV) [32,36,44]. Three of the studies used AD8 cut off score ≥2 [32,45,46], and two of the studies ≥3 [36,44]. The number of participants in the studies was between 109 and 10,340, in total 13,345. One study included participants ≥60 years of age [44], three studies ≥65 [36,45,46] and one study ≥75 years[32].

### Methodological quality

The assessment of methodological quality in accordance with the QUADAS-2 is summarised in Table 2. Information about time elapsing between the performance of index test and further evaluation in the three studies having more than one visit was poorly reported [32,36,44]. In the study by Meguro et al. [32], with a 2-year data collection time, this was assessed as having an unclear risk of bias. In the study by Correia et al. [36], the study sample was population-based; however, patient selection was performed by a trained census or a community health agent working in the area, which may have induced bias. High drop out in the study by Chan et al. [44] may have had an effect on prevalence, thereby leading to concerns regarding applicability in patient selection. In the study by Yang et al. [46], information about the informants was missing, thus leading to concerns of bias within the index test. The other domains of all five studies were assessed as having a low risk of bias and applicability.

**Table 2.** Quality assessment of included studies.

Study	Risk of bias				Applicability concerns		
	Patient selection	Index test	Reference standard	Flow and timing	Patient selection	Index test	Reference standard
Chan et al. [44]	Low	Low	Low	Low	Unclear	Low	Low
Correia et al. [36]	Unclear	Low	Low	Low	Low	Low	Low
Mao et al. [45]	Low	Low	Unclear	Low	Low	Low	Low
Meguro et al. [32]	Low	Low	Low	Unclear	Low	Low	Low
Yang et al. [46]	Low	Unclear	Unclear	Low	Low	Low	Low

## Discussion

The results of this systematic review show that the AD8 has a greater ability to predict people who are at risk of not having dementia (NPV: 96–100%) than to correctly identify those at risk of having dementia (PPV: 28–63%) within the target population. However, with a sensitivity ranging between 88–100% – indicating the AD8 has a good ability to identify the true positives – the lower specificity (67–91%) indicates the statistical risk of AD8’s ability to also identify false positives. Consistent with the Grading of Recommendations Assessment, Development and Evaluation working group [47], the quality of evidence in this systematic review can be discussed both from the DTA variables as individual outcome measures and as an overall assessment.

As the AD8 is an informant-based questionnaire and not a diagnostic test, the number of false positives will not get a misclassified dementia diagnosis but will be offered further evaluation, despite having dementia. The consequences of identifying persons with cognitive functional decline but who do not fulfil the diagnostic criteria of dementia can be discussed partly in the light of people with MCI. The estimated incidence rate of MCI within the target population per 1000 person-year in the age group 75–79 years is 22.5% with an increase to 60.1% for the age group 85 + [48]. In a Swedish cohort with the inclusion of the age group 50–79 years, 16% of 292 persons with self-observed or informant-reported cognitive decline at baseline had converted to dementia in the 2-year follow up and another 24% in the 6-year follow up [49]. Consequently, MCI can be seen as a prodromal state of dementia; however, an overall reversion rate to normal cognition of 18% has been seen [50]. Thus, persons with cognitive impairment are at greater risk for developing dementia and might benefit from early interventions such as coordinated care and management of symptoms, thereby resulting in, for example, cost savings, improved patient safety and postponement of institutionalizations [3,51]. However, the cognitive decline may also be caused by other medical, psychological or social conditions that are important to identify.

In the study by Chan et al. [44], the DTA improved when extracting ≥75 age group, indicating that a better PPV due to higher prevalence reduces false positives. This highlights the importance of evaluating the DTA within its context of use. The prevalence rate of included studies ranging from 8.9 to 22% and inclusion of participants from ≥60 to ≥75 shows a heterogeneity that is not suitable for meta-analysis in a disease where high age is the strongest risk factor. Also, as with all questionnaires, results are at risk of reporting bias. Studies have shown that a two-staged serial combination – with an informant questionnaire in stage one and an objective cognitive patient assessment instrument in stage two – can increase the DTA, especially in terms of increased specificity [52,53], which thus means lowering the number of false positives. This indicates a need for a larger toolbox that enables the case findings to be individually adapted.

Within a Swedish primary healthcare context, the AD8 would have the role of an initial screening instrument in identifying people with symptomatic dementia that still are unrecognised by the healthcare system and eligible for basic cognitive evaluation. In addition to being valid and reliable, a screening test should be easy to administer, inexpensive and do no harm [10]. Though the results from this systematic review show that the AD8 has a good ability to identify true positives, false negatives and predict people with low risk of having dementia within the target population, some limitations need to be considered. Only five studies were included. Even if the search strategy was developed in accordance with recommendations from the method literature and database-specific subject headings as well as free-text terms and manual searches of reference lists being used to find relevant studies, some studies might have been missed. Moreover, all five included studies were assessed as having an unclear risk of bias within one domain each, which may have had a greater estimated impact than the current assessment.

In conclusion, included studies show an overall low risk of publication bias and applicability concerns. The high sensitivity and NPV in all included studies show consistency, despite differences in reference standard, cut-off, age groups and size. However, the distribution in specificity and PPV includes a risk of identifying false positives, which must be further evaluated within its context of use. With a number of approximately 75,000 persons with symptomatic dementia in Sweden today still unrecognised by the health care system, an instrument for initial indicated screening may be of benefit for both the patients, their relatives and the healthcare system. The result of this systematic review shows that the informant-based questionnaire, AD8, has the potential to lower this gap, thereby allowing healthcare to provide better and safer healthcare for persons with symptomatic dementia and their caregivers.

## Appendix 1 Electronic search strategy, example from PubMed

**Table:**

	Search terms		Items found 2019-06-27	Items found 2019-11-20
# 1	AD8	Index test	203	209
# 2	AD-8		119	122
# 3	‘Ascertain dementia 8 questionnaire’		45	45
# 4	‘Ascertain dementia eight questionnaire’		6	6
# 5	‘Eight-item Informant Interview to Differentiate aging and Dementia’		1	1
# 6	‘Eight-item Interview to Differentiate aging and Dementia’		4	4
# 7	‘AD8 Dementia Screening Interview’		21	21
# 8	‘The Alzheimer Disease-8’		4	4
# 9	‘The Alzheimer’s Disease-8’		12	12
# 10	#1 OR #2 OR #3 OR #4 OR #5 OR #6 OR #7 OR #8 OR #9		371	380
# 11	Dementia	Outcome	197,030	202,644
# 12	Neurocognitive disorders [MESH]		237,465	243,827
# 13	Cognitive		375,452	389,165
# 14	#11 OR #12 OR #13		554,938	572,335
# 15	#10 AND #14	Combined sets	186	193
# 16	2005-08-01 to 2019-06-27	Limits	153	159

## Appendix 2. Description of included studies

**Table:**

Study; author; year/country	Setting; age; size; inclusion/exclusion criteria	Index test language/cut off; reference standard; outcome test accuracy	Study quality
Clinical Utility of the Informant AD8 as a Dementia Case Finding Instrument in Primary Healthcare; Chan QL, Xu X, Shaik MA, Chong SS, Hui RJ, Chen CL, et al. [44]; 2016/Singapore	Primary health care centers; Age: 71.7 ± 8.2; Size: 309; Inclusion: ≥60, physical ability to perform cognitive assessments and having an informant.	Chinese-Malay / ≥3; DSM IV; Dementia (CDR ≥ 1.0); Sensitivity: 91 % Specificity: 91 % Prevalence: 14.2 %	Patient selection: Random sampling at two primary health care centers. Of 3135 asked for participation 1082 consented for phase 1. Of 1076 eligible for phase 2, 309 consented and completed evaluation. Analyses show no difference in AD8 results but higher age, more female, lower education in the drop-outs between phase 1 and 2. Index test: Cut-off ≥ 3 prespecified. Result of reference standard blinded. Serial testing with AD8 in phase 1 as screening. Reference standard: Result of index test blinded. Phase 2 including clinical and neuropsychological evaluation at a research center. Flow and timing: All participants evaluated towards reference standard. Data collection between November 2013 and August 2014. Information missing of time elapsing between index test and further evaluation. Quality assessment: Moderate
AD8-Brazil: Cross-Cultural Validation of the Ascertaining Dementia Interview in Portuguese; Correia CC, Lima F, Junqueira F, Camos MS, Bastos O, Petribú K, et al. [36]; 2011/Brazil	Community dwelling; Age: 76.7 ± 0.6 (female); Size: 109; Inclusion: ≥65 and an informant who knew them for at least 10 years.	Brazilian-Portuguese / ≥3; DSM IV; Dementia (CDR ≥ 1.0); Sensitivity: 100 % Specificity: 67 % Prevalence: 13.8 %	Patient selection: Calculated sample size of 101 was achieved with 109 participants of 111 asked for consent. Participants identified and recruited by trained census or community health agent working in the area. Index test: Serial testing with index test as part of phase 1.Result of reference standard blinded. Cut-off ≥ 3 prespecified. Reference standard: Result of index test blinded. Phase 2 was performed by geriatrician or psychiatrist. Flow and timing: All participants evaluated towards reference standard. Data collection between October 2008 and January 2009. Information missing of time elapsing between index test and further evaluation. Quality assessment: Moderate
Diagnostic accuracy of Instrumental Activities of Daily Living för dementia in community-dwelling older adults; Mao HF, Chang LH, Tsai AY, Huang WW, Tang LY, Lee HJ, et al. [45]; 2018/Taiwan	Community-dwelling; Age: 74.87 ± 6.03; Size: 10,340; Inclusion: ≥ 65, not living in institution.	Chinese / ≥2; NIA-AA; Dementia (CDR ≥1.0); Sensitivity: 88 % Specificity: 84 % Prevalence: 8.9 %	Patient selection: Door-to-door survey. With the aim of 12,500 participants, 28,600 were randomly sampled. 10,571 agreed to participate, 10,340 completed evaluations. Participants representative according to analyses. Index test: Parallel testing of three tests. All included as part of evaluation towards reference standard. Cut-off score pre-specified. Reference standard: Result of index test part of the evaluation. Performed by trained nurses at one visit. Flow and timing: All participants evaluated towards reference standard. Data collection between December 2011 and March 2013. Quality assessment: High
Reliability and Validity of the Japanese Version of the AD8; Meguro K, Kasai M, Nakamura K. [32]; 2015/Japan	Community-dwelling; Mean age: 80.1; Size: 572; Inclusion: ≥75	Japanese / ≥2; DSM IV; Dementia (CDR ≥ 1.0); Sensitivity: 88.4 % Specificity: 68.4 % Prevalence: 12 %	Patient selection: Community resident*s* ≥ 75 living in Kurihara, Northern Japan. No selection. 590 of 1252 agreed to participate. 18 was excluded due to incomplete completion of tests. Mean age 82.2 among no-agreements. Index test: Result of reference standard blinded. Cut-off score not pre-specified due to validation study. Reference standard: Serial evaluation conducted independently by nurse and doctor. Flow and timing: All participants were evaluated towards reference standard. Data collection between 2008-2010. Information missing of time elapsing between index test and further evaluation. Quality assessment: Moderate
Screening for Dementia in Older Adults: Comparison of Mini-Mental State Examination, Mini-Cog, Clock Drawing Test and AD8; Yang L, Yan J, Jin X, Jin Y, Yu W, Xu S, et al. [46]; 2016/China	Community-dwelling; Age: 79.5 ± 7.6; Size: 2015; Inclusion: ≥ 65, ability to perform Clock Drawing Test, not being critically ill.	Chinese / ≥2; NIA-AA; Dementia (MMSE < 20/23/27); Sensitivity: 89.6 % Specificity: 78.4 % Prevalence: 22 %	Patient selection: All community-dwellin*g* ≥ 65 in a province in eastern China selected through multi-stage stratified random cluster sampling was invited to participate. 2063 agreed, 45 were excluded. Prevalence 13 % when adjusted to standard population. Index test: Result of reference standard blinded. Cut-off score pre-specified. Parallel testing performed by psychiatrist. Information missing about informants. Reference standard: Physical evaluation by nurse and testing by psychiatrist. Flow and timing: All participants evaluated towards reference standard. Data collection between May 2014 and November 2014. Quality assessment: Moderate

## Appendix 3. Accuracy calculations of the AD8 for identifying dementia in included studies

**Table:**

Study	Dementia	Non-dementia
	Sensitivity: (TP/(TP + FN))	Specificity: (TN/(FP + TN))
Chan et al. [44]	40/(40 + 4) = 0.909	241/(24 + 241) = 0.909
Correia et al. [36]	15/(15 + 0) = 1.00	63/(31 + 63) = 0.670
Mao et al. [45]	804/(804 + 113) = 0.877	7979/(1464 + 7979) = 0.845
Meguro et al. [32]	61/(61 + 8) = 0.884	344/(159 + 344) = 0.684
Yang et al. [46]	398/(398 + 46) = 0.896	1232/(339 + 1232) = 0.784
	PPV: (TP/(TP + FP))	NPV: (TN/(FN + TN))
Chan et al. [44]	40/(40 + 24) = 0.63	241/(4 + 241) = 0.98
Correia et al. [36]	15/(15 + 31) = 0.33	63/(0 + 63) = 1.00
Mao et al. [45]	804/(804 + 1464) = 0.35	7979/(113 + 7979) = 0.99
Meguro et al. [32]	61/(61 + 159) = 0.28	344/(8 + 344) = 0.98
Yang et al. [46]	398/(398 + 339) = 0.54	1232/(46 + 1232) = 0.96
	Accuracy: (TP + TN/(TP + FP + FN + TN)	
Chan et al. [44]	(40 + 241)/309 = 0.91	
Correia et al. [36]	(15 + 63)/109 = 0.72	
Mao et al. [45]	(804 + 7979)/10340 = 0.85	
Meguro et al. [32]	(61 + 344)/572 = 0.71	
Yang et al. [46]	(398 + 1232)/2015 = 0.81	
	LR+ = (sensitivity/(1 − specificity))	LR− = ((1 − sensitivity)/specificity)
Chan et al. [44]	0.909/(1 − 0.909) = 9.99	(1 − 0.909)/0.909 = 0.10
Correia et al. [36]	1.0/(1 − 0.670) = 3.03	(1 − 1.0)/0.670 = 0.0
Mao et al. [45]	0.877/(1 − 0.845) = 5.66	(1 − 0.877)/0.845 = 0.15
Meguro et al. [32]	0.884/(1 − 0.684) = 2.80	(1 − 0.884)/0.684 = 0.17
Yang et al. [46]	0.896/(1 − 0.784) = 4.15	(1 − 0.896)/0.784 = 0.13

TP: true positive; FP: false positive; FN: false negative; TN: true negative; PPV: positive predictive value; NPV: negative predictive value; LR+: positive likelihood ratio; LR−: negative likelihood ratio.
